# Glucagonoma syndrome: a case report

**DOI:** 10.1186/1752-1947-5-402

**Published:** 2011-08-22

**Authors:** Pablo Granero Castro, Alberto Miyar de León, Jose Granero Trancón, Paloma Álvarez Martínez, Jose A Álvarez Pérez, Jose C Fernández Fernández, Carmen M García Bernardo, Luis Barneo Serra, Juan J González González

**Affiliations:** 1 Department of General Surgery and Gastroenterology, Hospital Universitario Central de Asturias, Oviedo, Spain

## Abstract

**Introduction:**

Glucagonoma syndrome is a rare paraneoplastic phenomenon, with an estimated incidence of one in 20 million, characterized by necrolytic migratory erythema, hyperglucagonemia, diabetes mellitus, anemia, weight loss, glossitis, cheilitis, steatorrhea, diarrhea, venous thrombosis and neuropsychiatric disturbances in the setting of a glucagon-producing alpha-cell tumor of the pancreas. Necrolytic migratory erythema is the presenting manifestation in the majority of cases, so its early suspicion and correct diagnosis is a key factor in the management of the patient.

**Case presentation:**

We present the case of a 70-year-old Caucasian woman with glucagonoma syndrome due to an alpha-cell tumor located in the tail of the pancreas, successfully treated with surgical resection.

**Conclusion:**

Clinicians should be aware of the unusual initial manifestations of glucagonoma. Early diagnosis allows complete surgical resection of the neoplasm and provides the only chance of a cure.

## Introduction

A glucagonoma is a slow-growing alpha-cell tumor of the pancreatic islets of Langerhans. It may appear as a benign and localized alpha-cell adenoma but at least 50% of cases will have metastatic disease when diagnosed [1]. Glucagonomas can be associated with other tumors in Multiple Endocrine Neoplasia syndrome 1 (MEN 1), but this association is rare and comprises no more than 3% of glucagonomas. Even though glucagonomas related to MEN 1 syndrome probably carry a better prognosis due to early recognition through periodic screening visits, 80% are malignant and frequently spread to the liver [2]. Glucagonoma syndrome is a rare paraneoplastic phenomenon, with an estimated incidence of one in 20 million, characterized by necrolytic migratory erythema (NME), hyperglucagonemia, diabetes mellitus, anemia, weight loss, glossitis, cheilitis, steatorrhea, diarrhea, venous thrombosis and neuropsychiatric disturbances in the setting of a glucagon-producing alpha-cell tumor of the pancreas [3]. The most common features of this syndrome are weight loss, NME and diabetes mellitus [4]. Of these, NME presents as the hallmark clinical sign of glucagonoma syndrome [3]. Its early recognition allows a prompt diagnosis of the tumor and leads to a better prognosis. Surgery is the optimal treatment for a glucagonoma. We present a patient with glucagonoma syndrome due to a well circumscribed alpha-cell tumor of the pancreas, in which surgical removal of the tumor by distal pancreatectomy with splenectomy led to resolution of the cutaneous and systemic features.

## Case presentation

**Figure 1:**
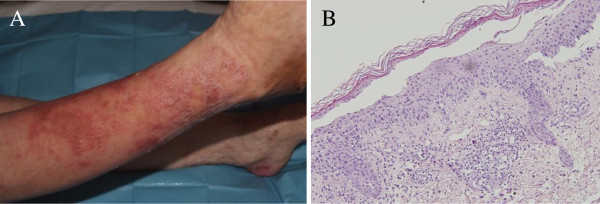
**Necrolytic migratory erythema.** (A) Skin lesions affecting pretibial area. (B) Skin biopsy in necrolytic migratory erythema showing a zone of necrolysis and vacuolated keratinocytes.

**Figure 2:**
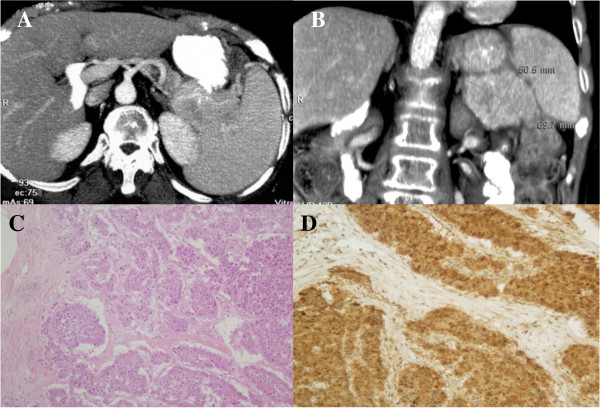
**Radiological and histological findings.** (A) Axial and (B) coronal CT scan revealing a 5-7 cm nodular mass in the tail of her pancreas (*)(C) Histological examination of the mass showing an alpha-cell pancreatic tumor (hematoxylin and eosin ×20). (D) Inmunostaining revealed numerous glucagon-positive cells (×20).

A 70-year-old Caucasian woman was referred to our Department of Dermatology with a persistent subacute eczema affecting her lower extremities and groin area that had been present for 12 months. She was treated with topical and oral steroids with no improvement. Her medical history revealed a long-standing type 2 diabetes mellitus and recurrent episodes of deep-vein thrombosis in her right leg despite anticoagulant therapy. The skin eruption initially appeared in her lower extremities but there was a rapid progression with involvement of her trunk, upper extremities and perioral area. These skin lesions were associated with weight loss (15 kg in one year), anorexia, weakness, glossitis and angular stomatitis. A physical examination revealed itching cutaneous eruptions of erythematous polycyclic migratory lesions with scaling advancing borders and central resolution. The entire course of the local skin lesion healed within two weeks while new cutaneous eruptions occurred in other locations. Chronic lesions often evolved into lichenification. Laboratory data showed a low hemoglobin level (10.5 g/dL), hyperglycemia (176 mg/dL), hypoalbuminemia (22 g/L) and hypoproteinemia (49 g/L). Her white cell count (6100/μL) and platelet level (26.2 × 10^4^/μL) were also within normal limits, and an abnormality of cell form was not found in her peripheral blood. Her levels of serum iron, vitamin B12 and erythropoietin, and the number of reticulocytes were found to be normal. Electrophoresis of her serum protein was performed because of the possibility of multiple myeloma; however, no abnormal protein was found. Although the possibility of gastrointestinal (GI) bleeding was considered, no abnormality was detected on an upper GI endoscopy, barium enema, or barium examination of her small bowel. A skin biopsy performed on a peritibial lesion showed a spongiotic epidermis with vacuolization of the granular layer and presence of necrotic keratinocytes in the horny layer. A mild infiltrate of lymphocytes was present in the papillary dermis (Figure 1). Histological findings were compatible with the diagnosis of NME. Ultrasonography was performed as a screening examination, and revealed a hypoechoic tumor in her distal pancreas. An abdominal computed tomography (CT) scan showed a hypervascularized tumor measuring 5 to 7 cm in the tail of her pancreas without evidence of metastatic disease (Figure 2). Carcinoembryonic antigen and carbohydrate antigen 19-9 levels were normal. The exocrine function of her pancreas was normal. However, her level of serum glucagon was elevated to 2340 pg/mL (normal range, 55-177 pg/mL), while her levels of other hormones, such as somatostatin or gastrin, were within normal limits, and insulin was low. Glucagonoma of the pancreas was diagnosed and distal pancreatectomy with splenectomy was performed. This resection involved dissection of her regional lymph nodes (D1). Histopathological examination revealed a 6 cm alpha-cell pancreatic tumor with vascular and perineural tumor invasion. Dissection of the regional lymph nodes showed that a total of 16 lymph nodes were isolated, of which three were affected. Inmunohistochemical staining was positive for glucagon, chromogranin and synaptophysin, but negative for other hormones, such as insulin, gastrin and somatostatin. On the basis of these findings, a diagnosis of malignant glucagonoma of the pancreas was made. Five days after surgery, the skin lesions disappeared and postoperative plasma glucagon levels decreased to 197 pg/dL. Our patient has been without recurrence for one and a half years since the surgery and remains asymptomatic.

## Discussion

Stacpoole [5] reported that all of the following criteria should be satisfied to diagnose a glucagonoma: demonstration of a tumor mass by direct visualization or radiographic techniques; proof that the tumor shows a preponderance of glucagon-containing cells on appropriate staining and/or proof of increased tissue levels of immunoreactive glucagon; elevation of basal circulating immunoreactive glucagon; and at least one of the following coincidental findings; (a) skin rash, (b) glucose intolerance, or (c) hypoaminoacidemia. The patient reported here fulfilled these criteria.

The high level of glucagon secreted by the tumor may promote glycogenolysis, gluconeogenesis, ketogenesis and lipodieresis by activating phosphorylase in the liver, stimulating the secretion of insulin, and inhibiting the external secretion of the pancreas, thus resulting in the increased level of blood glucose. In persistent hyperglucagonemia, diabetes mellitus develops at the expense of tissue glycogen stores, muscle and fat mass. Impaired fasting glycemia or diabetes mellitus is found in 80% of patients with glucagonoma syndrome [6]. Hyperglucagonemia in healthy patients and in glucagonoma syndrome reduces plasma amino acid concentrations and enhances essential amino acid catabolism.

NME is the presenting manifestation in 70% of patients with glucagonoma syndrome [3]. The lesions consist of erythematous scaling and crusting patches most frequently observed in the groin, intergluteal and genital areas. Central healing may occur giving an annular appearance. The most specific feature on skin histological examination is necrolysis of the upper epidermis with vacuolated keratinocytes, leading to focal or confluent necrosis, but this histopathologic feature may be seen in other deficiency states like pellagra, necrolytic acral erythema or zinc deficiency [4]. Normalization of glucagon concentrations by surgery results in a rapid disappearance of the skin rash. However, abnormal glucagon levels alone cannot explain all of the skin findings. Hypoaminoacidemia, nutritional lack of zinc and fatty acids or hepatocellular dysfunctions are all considered to be possible triggering factors of NME. Hyperglucagonemia provokes multiple nutrient and vitamin B deficiencies, which in turn are the probable cause of this typical skin disorder [7]. Other systemic pathologies, such as chronic liver disease, inflammatory bowel disease, malabsorptive state, pancreatitis, various malignant neoplasms and heroin abuse have been associated with NME without glucagonoma. The early recognition of NME is therefore important because it will prevent the catabolic clinical features and reduce the risk of metastasis with obvious quality of life improvements. Even though NME has traditionally been considered an early manifestation of disease, it is more likely a late manifestation of years of tumor growth. The relatively low occurrence rate of NME in patients with MEN-associated glucagonoma diagnosed early in the clinical course by screening efforts supports this contention [3,8].

As seen in our patient, glucagonoma syndrome can be associated with a high incidence of thromboembolism. A thromboembolic phenomenon, such as deep-vein thrombosis or pulmonary embolism, may be present in 10-30% of patients, often resulting in death. In fact, thromboembolic events may account for over 50% of all deaths directly attributed to the glucagonoma syndrome. The mechanism for this coagulopathy is poorly understood and seems to be related to an increased factor × production by the pancreatic alpha-cells [9].

Although the optimal treatment for glucagonoma is surgery, 50% of the tumors have metastasized by the time of diagnosis [1]. Transabdominal ultrasound has been used to demonstrate tumor localization but has limited utility because of its inferior sensitivity. Endoscopic ultrasound visualization of the tumor is reportedly highly sensitive but has not gained widespread acceptance. A contrast-enhanced CT scan is very useful in attempting to demonstrate the presence of a pancreatic tumor and it is usually the initial radiographic test because of its non-invasiveness. However, selective visceral angiography is considered the gold standard in diagnosis and localization of glucagonomas. Its superior sensitivity is related to the hypervascularity of these neoplasms. Although it is an invasive test, it has the advantage of being able to demonstrate hepatic metastasis even in cases with normal liver scans. The role of magnetic resonance imaging (MRI) in the diagnosis of glucagonoma has not been clearly defined. The utility of pancreatic venous sampling for glucagon levels to diagnose smaller tumors has also been reported [5]. The diagnosis is made by the finding of a pancreatic alpha-cell tumor. Although the average size of a glucagonoma may be large, diagnostic confirmation and localization by needle biopsy is not usually performed due to the ease and precision of alternative methods. These other methods to localize the tumor in suspected cases include ultrasound, CT and selective visceral angiography. Tumors and metastases can be visualized by CT. Neuroendocrine tumors, in contrast to pancreatic exocrine adenocarcinoma, are hypervascular lesions, and this characteristic is often useful when reviewing imaging studies. Somatostatin receptor scintigraphy is frequently used as a complementary method to conventional imaging such as CT and MRI as it is useful for consistent supervision of somatostatin receptor expression and dissemination of the tumor metastases [4,10]. The liver is the most frequent site of metastasis, followed by the peripancreatic lymph nodes, bone adrenal gland, kidney and lung.

Pancreatic endocrine tumors represent a heterogeneous group with varying tumor biology and prognosis. These neoplasms are classified as functional if they are associated with a hormone-related clinical syndrome caused by hormone release from the tumor, or non-functional if the tumor is not associated with a hormone-related clinical syndrome [11]. The differential diagnosis is based on histopathology demonstrating neuroendocrine features such as positive staining for chromogranin A and specific hormones such as gastrin, proinsulin and glucagon. The differential diagnosis of glucagonoma includes other primary pancreatic neoplasms, intrapancreatic malignant mesothelioma, and solid pseudopapillary neoplasms [12]. A close relationship between glucagon expression in pancreatic endocrine tumors and cystic formation is also reported in the literature, but cystic glucagonomas are not associated with a glucagonoma syndrome in the majority of cases as they are non-functioning glucagon-producing neuroendocrine tumors [13].

Treatment of glucagonoma syndrome should be directed at the underlying etiology. Removal of the primary tumor with a distal pancreatectomy brought evident relief of all clinical symptoms for 1 to 2 year periods. Pancreatic fistula and delayed gastric emptying are the most prevalent complications of distal pancreatectomy but they can be managed by conservative measures in the majority of cases. The tumor is resistant to chemotherapy and metastatic disease is often not amenable to surgical resection. The prognosis of this disease varies greatly according to the stage at which the disease is diagnosed. Estimations of mean survival after diagnosis have ranged from three to seven years or more [14]. Long-acting somatostatin analogues, which are potent inhibitors of glucagon release, have been proven effective in suppressing glucagon secretion from glucagonomas and controlling the metastatic growth. Since the tumor is slow growing, prolonged survival is possible and, in metastatic disease, most causes of death appear to be unrelated to the tumor. Control of liver metastases by metastasectomy, cryoablation, radiofrequency ablation or chemoembolization has been reported [4,6]. Recent studies suggest that conventional contraindications to surgical resection, such as superior mesenteric vein invasion and nodal or distant metastases, should be redefined in patients with advanced neuroendocrine tumors. These patients will benefit from extensive surgical debulking in combination with adjuvant medical treatments, such as somatostatin analogues. This combination may result in enhanced survival rates compared with either procedure alone [10].

## Conclusion

Clinicians should be aware of the unusual initial manifestations of glucagonoma. Early diagnosis allows complete surgical resection of the neoplasm and provides the only chance of a cure.

## Consent

Written informed consent was obtained from the patient for publication of this case report and any accompanying images. A copy of the written consent is available for review by the Editor-in-Chief of this journal.

## Competing interests

The authors declare that they have no competing interests.

## Authors' contributions

AML, JGT, PAM, JAP, JFF, CGB, LBS and JGG were involved in the direct care of this patient. In addition, PGC was responsible for drafting the manuscript and JAP, JGT and PAM helped to draft the manuscript. All authors have read and approved the final manuscript.
